# Medically induced amenorrhea in female astronauts

**DOI:** 10.1038/npjmgrav.2016.8

**Published:** 2016-04-21

**Authors:** Varsha Jain, Virginia E Wotring

**Affiliations:** 1 National Institute for Health Research, London, UK; 2 King's College London, London, UK; 3 Queen Mary's University, London, UK; 4 Center for Space Medicine, Baylor College of Medicine, Houston, TX, USA; 5 Department of Pharmacology, Baylor College of Medicine, Houston, TX, USA

## Abstract

Medically induced amenorrhea can be achieved through alterations in the normal regulatory hormones via the adoption of a therapeutic agent, which prevents menstrual flow. Spaceflight-related advantages for medically induced amenorrhea differ according to the time point in the astronaut’s training schedule. Pregnancy is contraindicated for many pre-flight training activities as well as spaceflight, therefore effective contraception is essential. In addition, the practicalities of menstruating during pre-flight training or spaceflight can be challenging. During long-duration missions, female astronauts have often continuously taken the combined oral contraceptive pill to induce amenorrhea. Long-acting reversible contraceptives (LARCs) are safe and reliable methods used to medically induce amenorrhea terrestrially but as of yet, not extensively used by female astronauts. If LARCs were used, daily compliance with an oral pill is not required and no upmass or trash would need disposal. Military studies have shown that high proportions of female personnel desire amenorrhea during deployment; better education has been recommended at recruitment to improve uptake and autonomous decision-making. Astronauts are exposed to similar austere conditions as military personnel and parallels can be drawn with these results. Offering female astronauts up-to-date, evidence-based, comprehensive education, in view of the environment in which they work, would empower them to make informed decisions regarding menstrual suppression while respecting their autonomy.

## Introduction

Physiological mechanisms during the natural menstrual cycle involve a coordinated interplay among regulatory hormones. Hypothalamic release of gonadotropin-releasing hormone stimulates the pituitary gland to produce follicle-stimulating hormone and luteinizing hormone, which peaks mid-cycle. This peak invokes ovulation. The developing ovum produces estrogen and its remaining ‘shell’, i.e., the corpus luteum produces progesterone. Endometrial thickening commences in preparation for a pregnancy; however, when fertilization does not occur, estrogen and progesterone levels decrease causing endometrial shedding. This is released cyclically as menstrual flow.

Medically induced amenorrhea is the adoption of a therapeutic device (e.g., levonorgestrel intrauterine device (LNG-IUD)) or treatment (typically hormonal preparations, e.g., the combined oral contraceptive (COC) pill or depot medroxyprogesterone acetate (DMPA)) that act on part or all of the above mechanisms in order to prevent menstrual flow. Routinely, 21 COC pills are taken daily; these contain active ingredients that suppress ovulation and thin the endometrium. Then, for the next 7 days, either a break is taken from the active ingredient pills or placebo pills are taken, and during this time a withdrawal bleed occurs. This differs from a menstrual bleed. Medically induced amenorrhea would also include the delay or suppression of this withdrawal bleed.

Modern women living in an industrialized country have more menstrual cycles compared with women of pre-historic times. There are an estimated 450 ovulations per lifetime now, compared with 160 ovulations potentially due to later menarche, earlier first births, frequent closely spaced pregnancies, long periods of breastfeeding and living shorter lives.^1^ The 21-day treatment/7-day placebo COC cycle was developed in the 1960s to mimic a natural cycle and increase adherence with a daily pill. Thoughts on whether women need to menstruate every month vary widely and have cultural determinants^2,3^ but menstrual suppression is gaining favor and becoming more common. Physicians’ attitudes to medically induced amenorrhea also vary and may affect long-term acceptance.^4^ The side-effect profiles for menstrual suppression regimes are grossly similar to when the same agents are used for contraception (Table 1) and return to fertility occurs with treatment cessation with most agents.^5^

## Advantages of menstrual suppression

### General

There are numerous reasons for menstrual cycle control. Arresting cycles can alleviate or improve medical disorders, e.g., gynecological problems such as menorrhagia or endometriosis, hematologic conditions including inherited bleeding disorders, and neurologic disease such as menstrual headaches.^6^ Menstrual cycles can also be suppressed on a short- or long-term basis for convenience, e.g., during exams, for special holidays, or after tubal ligation for sterilization. Women can control their cycles according to personal circumstances and convenience.

### Spaceflight related

Individuals in austere conditions (deployed military personnel or astronauts) may welcome amenorrhea, with advantages beyond those sought in the traditional clinic setting.

**Figure 1:**
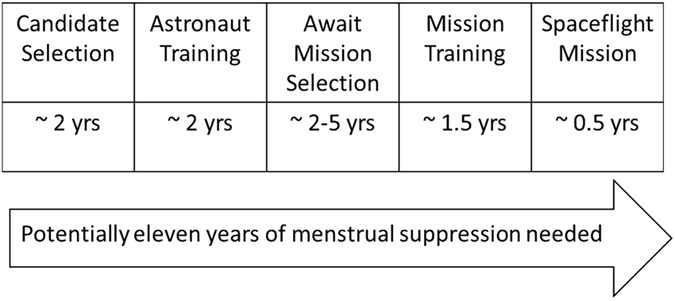
Approximate time over which menstrual suppression±contraception may be required by female astronauts.

Pregnancy can delay aspects of astronaut selection and training. It is contraindicated for pre-flight training activities, including vacuum chamber exercises or high-performance jet flying, and spaceflight. With a rigorous pre-flight training phase, including frequent international travel followed by time in quarantine before missions, lasting at least 6 months, this further increases the time over which reliable and effective contraception is important (Figure 1). During the shuttle era, there were concerns about pre-flight missed pills and therefore the risk of possible pregnancy. There are, however, no cases where pregnancy prevented an astronaut flying her designated mission.^7^ The ideal contraceptive method would therefore help ensure effective amenorrhea as well as higher adherence rates in order to reduce pregnancy risk.

As well as contraceptive effects, menstrual suppression is an added benefit of some contraceptive methods. The waste disposal systems onboard the US side of the International Space Station that reclaim water from urine were not designed to handle menstrual blood, thus idealizing the minimization of breakthrough bleeding during menstrual suppression. The practicalities of personal hygiene while menstruating during spaceflight could be challenging, e.g., limited wash water supply or the task of changing hygiene products in microgravity. Nonetheless, full amenities are available should astronauts choose to menstruate. It is more common for astronauts to continually suppress their cycles for long-duration missions compared with short-duration missions (Jennings, R.T., oral communication, 12 November 2014). Short-duration missions allowed the flexibility for menstrual cycles to be timed according to mission dates, thus avoiding menstruation in space as well as the need for menstrual suppression (Baker, E.S., oral communication, 29 October 2014); female astronauts could time shift their cycles with hormonal therapy in advance of a mission. This cannot be done with long-duration missions and therefore the question arises as to whether the female astronaut wishes to suppress or not suppress.

## Menstrual suppression methods for astronauts

### Options for female astronauts

Terrestrially, women may suppress menses via extended or continuous use of the daily COC pill, daily progesterone-only pills (POPs), the three monthly progestin-only injection DMPA, the progestin-based subdermal implant that is viable for at least 3 years, or the LNG-IUD that is currently licensed for 5 years. As well as a variance in their ability to induce amenorrhea, these methods are also liable to individual variability within their end user. Several studies have found a reduction in bone mineral density (BMD) with DMPA.^8^ Terrestrially, these losses may be recovered once the therapy is ceased, however, due to irreversible spaceflight-related bone changes, a treatment option that may impact BMD would not be acceptable for this subpopulation of women. Only 50% of women using the POP become anovulatory and the rest continue to menstruate regularly. Unless a woman is unable to use estrogen-containing products, the POP would not provide an astronaut with the desired rate of menstrual suppression or minimized breakthrough bleeding that would be needed. The progestin-based subdermal implant provides variable rates of amenorrhea, ~11–42%. These rates are lower than continuous COC use or the LNG-IUD; however, this device provides an additional long-acting reversible option for those not able to use estrogen or for those averse to having an intrauterine device. Therefore, continuous use of the COC, the subdermal implant, and the LNG-IUD appear to be the best options for menstrual suppression in the female astronaut population.

### Acceptability

For the astronaut population, their menstrual suppression regime has often involved continuous use of the daily COC for numerous years. Regimes used for COC can be tailored according to the woman’s needs. A Brazilian survey showed that continuous use of the COC was the more frequent prescribed method for inducing amenorrhea (79.4%) with the LNG-IUD following closely at 72.7%. It is important to note that patients requested and gynecologists suggested these forms of contraception in 81% of cases, with 86.2% specifically prescribed to induce amenorrhea.^9^

Long-acting reversible contraceptives (LARCs) are now available worldwide and provide a safe and reliable alternate method to continuous COC use for inducing menstrual suppression. LARCs encompass the progestin-containing subdermal implant and the LNG-IUD. Neither contain estrogen and therefore are not susceptible to estrogenic side effects or restrictions. They provide long-acting contraceptive benefits and are used medically to treat menorrhagia, fibroids, and endometriosis, whereas not impacting BMD. LARCs offer long-term therapeutic advantages as well as a reduction in mission upmass in comparison with daily COCs, no packaging to dispose, and they dispel concerns regarding stability during storage.

Despite the numerous advantages of using LARCs (outlined in Table 1), a US study evaluating contraceptive use terrestrially up until 2010 discovered that of the 62% of women using contraception in the study, only 5.6% used LARCs compared with 28% using COC and 27% who were sterilized.^10^ The percentage of women using LARCs is fortunately increasing and another study that evaluated the US National Survey of Family Growth data until 2013 highlighted a recent increase from 8.5% (2009) to 11.6% (2012) in overall LARC usage. This was predominately driven by intrauterine device (IUD) use, which rose from 7.7 to 10.3% between 2009 and 2012, rather than implant usage, which remained low.^11^ When no-cost contraception was offered to women aged 14–45 years in the contraceptive CHOICE project, LARCs were the preferred method of contraception. In addition to this, satisfaction with LARCs is high, demonstrated by 76.7% of LARC users still continuing with their contraceptive agent at 24 months, compared with 40.9% of non-LARC users. This trend was present in both the adolescent (14–19 years) and adult (20–45 years) groups.^12^ An uptake rate of 11.6% in 2012 therefore represents an underutilization of LARCs.

Better education for both physicians and patients could improve acceptability of these therapies. Recent military studies have shown continuous COC usage is only 15% in aviation personnel, despite operationally relevant benefits associated with its use.^13^ Military women desired to use menstrual suppression as an alternative to experiencing menstruation during deployment.^14^ Many women desired suppression for deployment (66%) but only 21% continuously used COCs. Of note, desire for mandatory education about continuous COC usage in this population was high, with 86% reporting this type of education should be an entry requirement for all female personnel.^15^ Knowledge gaps and compliance difficulties limit use for continuous COCs and therefore menstrual suppression in the military.^15^ A small UK military study highlighted the need for military healthcare providers to counsel personnel about contraceptive options rather than leaving this responsibility with national healthcare providers due to the higher levels of exposure to female soldiers. LARCs had not been discussed with large numbers of personnel, whereas contraceptive use was not even documented in over half of the consultation records in the year leading up to the study.^16^ The main barriers to uptake of LARCs include women’s knowledge of and attitudes towards the methods, practice patterns among providers, and high initial upfront costs. The contraceptive CHOICE project removed these barriers, and in turn found over two-third of participants chose a LARC (56% choosing an IUD and 11% selecting a subdermal implant).^17^

### Bone health with medically induced amenorrhea

The constant inhibition by oral synthetic estrogen and progesterone during continuous COC use leads to suppression of hypothalamic and pituitary release of follicle-stimulating hormone and luteinizing hormone.^18^ This in turn impacts bioavailable levels of estrogen and progesterone. Estrogen inhibits bone resorption through its actions on osteoclastic activity and has anabolic effects on osteoblasts.^19^ Continuous COC could therefore potentially benefit astronauts by reducing spaceflight-related osteopenia. It is however unknown how chronic low-dose COC therapy and its lack of peaks in estrogen delivery alongside the impact of microgravity together effect bone metabolism overall.

Elite athletes who are amenorrheic have been shown to have a lower BMD than those with regular cycles.^20^ Exercise is a vital countermeasure for astronauts and it is unknown if amenorrhea affects astronauts at the same rates as elite athletes. Mechanisms for osteopenic changes in relation to spaceflight are likely due to gravitational unloading as opposed to changes in the hypothalamic–pituitary–ovarian axis as is the case with elite athletes. It is unknown if there is a synergistic mechanistic action in some female astronauts who are extremely fit. Prescribing continuous COC in these athletic female astronauts to understand any potential advantages in relation to decreased BMD in space is also unknown.

Terrestrially, COCs do not appear to negatively affect BMD.^21^ However, low-dose (20 μg) ethinyl estradiol COCs have been shown to cause the loss of BMD compared with non-hormonal contraceptives (e.g., the copper IUD) at 3 years of use.^22^ The impact on overall BMD was particularly evident in adolescent subjects; however, a decrease was noted specifically in the femoral neck BMD in all pre-menopausal participants. The loss in BMD was small, even smaller than the effect of menopause; however, any BMD decrease within the astronaut population becomes relevant due to the irreversible bone changes that occur as a result of spaceflight. Little significance is given terrestrially to such small decreases due to risk versus benefit ratios, but the connotation of what may be ‘clinically relevant’, especially in terms of BMD, is different in the spaceflight realm.

The balance between estrogen and progesterone provided by hormonal contraception also needs consideration, as BMD was not lost in 30–35 μg of ethinyl estradiol COC users compared with 2 years continuous use of DMPA, which led to 6% BMD loss.^23^ Subjects in this study were physically fit and healthy as eligibility included being able to meet minimum criteria for entry into the armed forces. Over the course of the study, there was no requirement for regular physical exercise but the numbers that engaged in weight-bearing activity did not differ between experimental or control groups. Subjects were aged 18–33 years, therefore their bones were still under estrogenic influence; however, in the astronaut population, where bone preservation is vital, the potential advantages of oral estrogen cannot be ignored. Thus, COCs of 30–35 μg of ethinyl estradiol have been prescribed for continuous use among the astronaut population.^7^ Decisions regarding the dosage of estrogen within the COC were based on extrapolation from terrestrial studies, not from actual spaceflight evidence. There are currently no studies comparing the differences on bone loss in women who do or do not use the COC in space, and therefore this has not been formally evaluated.

It is important to note that the progestin-only subdermal implant and the LNG-IUD do not negatively impact BMD during usage on Earth in pre-menopausal women.^21^ This further increases their potential viability for female astronauts.

## Limitations of menstrual suppression

A potential limitation with virtually all menstrual suppression regimes is degree of suppression from initial dosing. Most studies quote rates of unscheduled, unpredictable and irregular bleeding in the initial months of LARC usage. It has been postulated that this could be attributed to the timing of initiation and whether the endometrium is in proliferative or secretory stages. There may also be an element of whether the hypothalamic–pituitary–ovarian axis is supporting ovulatory or anovulatory cycles.^6^

Weisberg *et al*.^24^ compared long-term bleeding patterns in LNG-IUD versus progestin-containing subdermal implant users. Irregular bleeding patterns are evident with all LARCs but amenorrhea rates appear to be similar in both the groups after 2 years of use. Transexamic acid and mefanamic acid have both been trialed to reduce the initial bleeding or spotting ‘nuisances’ with the LNG-IUD but neither led to a significant reduction compared with placebo in a double-blind randomized control trial.^25^ Evidence is limited around managing irregular bleeding with LNG-IUDs but with subdermal implants, non-steroidal anti-inflammatory drugs, or low-dose COCs may be trialed on a short-term basis in the United States.^26^ Trials in peri-menopausal women, where the LNG-IUD was used alongside oral estrogen therapy, have found high rates of amenorrhea, which are almost comparable with continuous COC use.^27^ Similarly, in the United Kingdom, up to 3 months of either continuous or cyclical 30–35 μg of ethinyl estradiol COCs are recommended to combat irregular bleeding for both LNG-IUDs and subdermal implants, although this is unlicensed.^28^

## Long-term effects of menstrual suppression

Long-term side-effect profiles for menstrual suppression by aforementioned methods are good. Data provide reassurance for the long-term use of COC pills and LARCs. Adverse events associated with extended regimes of COCs were similar to those seen with 28-day cyclical regimes.^29^ Return to a normal menstrual pattern and fertility is promising with both continuous COC use and LARCs. One-year pregnancy rate after the removal of the LNG-IUD is similar to women of the same age not using any form of birth control.^30^ Women discontinuing extended use COC regimes without starting any other hormonal contraception had a median time to withdrawal bleeding of 32 days, a return to ovulatory capacity within 32 days and 99% of women were having spontaneous menstruation or pregnancy within 3 months of discontinuation.^29^ There is no increased risk of breast cancer in users of the COC and it has been found that ovarian, endometrial, and colorectal cancer risks decrease as the length of COC use increases, i.e., making COC use protective against ovarian, endometrial, and colorectal cancer.^8,31^ The LNG-IUD is recommended for the treatment of endometrial hyperplasia and can be used long term with surveillance.^8^

## Operational considerations

From an operational perspective, LARCs would not be expected to interfere with the ability of the astronaut to perform her tasks. There are no reports in the literature suggesting high G loading experienced during launch or landing would impact subdermal implant or LNG-IUD placement or bleeding patterns; similar gravitational forces may be experienced by terrestrial women, e.g., military jet pilots but there are currently no data in the literature regarding this population. Perforation of the uterus or expulsion of the LNG-IUD is a risk associated with operator skill and more frequent in the first year of use. Should a LARC be desired (LNG-IUD or the subdermal implant), it would be advisable to insert it at least 1.5–2 years before flight, in order to judge the side-effect profile but more importantly the bleeding pattern. After the initial 6 months of use, bleeding patterns or probability of amenorrhea can be better judged with the LNG-IUD; however, bleeding patterns may differ with the subdermal implant. On the rare chance the LNG-IUD is expulsed during flight, menstruation would occur as normal during spaceflight and this would not impede an astronaut’s ability to perform her job. Ultrasound capabilities are already present onboard the International Space Station and a transvaginal ultrasound probe as well as gynecological examination kit could be added to the hardware if desired. Additional training as well as skill retention would need to be addressed before the implementation of such equipment.

Immune dysfunction may occur during spaceflight; however, this should not hinder an astronaut’s choice with regards to menstrual suppression options. Hormonal contraception is safe to use in immuno-compromised populations terrestrially, with COCs, subdermal implants, and LNG-IUDs having all been trialed. A pelvic inflammatory disease risk of 0.16 per 100 women years has been demonstrated within an immuno-compromised study group with IUDs, which is low.^32^ Earth-based analogs do not exist to precisely replicate spaceflight immune dysfunction but extrapolation from these data suggests continuous COCs and LARCs would be safe for female astronauts.

The daily requirement of the continuous COC regime would mean ~1,100 pills would be needed for a 3-year exploration class mission. Drug stability has not been tested for hormonal medications over such a long time in space or with the impact of deep-space radiation. Opting for a LARC would remove the cost, upmass, packaging, waste and stability issues as a device could be inserted before a mission and replacement would not be required in-flight. Consideration could be given as to whether a small number of 30–35 μg of ethinyl estradiol COC pills or non-steroidal anti-inflammatory drugs could be added to the medical kits in case breakthrough bleeding becomes problematic.

However, consideration may need to be given to the subdermal implant and whether it could rub or catch on specialist equipment or attire such as the Neutral Buoyancy Laboratory diving suit or the extra-vehicular activity suit. The implant does not usually interfere with normal clothing worn by terrestrial users, and most comment they are unaware of its positioning after the initial insertion. We suspect subdermal implant positioning would be non-problematic for female astronauts; however, this has not been trialed.

## Discussion

There is a long history of continuous COC use during spaceflight missions and training. It is safe and reliable for effective contraception and menstrual suppression. Other methods could be considered in the astronaut population, specifically LARCs, which are dependable and effective long-acting contraceptives with comparable rates of amenorrhea. The implant provides better rates of amenorrhea compared with the LNG-IUD in the first two years of use however thereafter rates of amenorrhea do not differ significantly. With the LNG-IUD currently licensed for five years versus three years for the implant, our recommendations would lie with the LNG-IUD due to time scales over which astronauts may require medically induced amenorrhea.

Treating bleeding irregularities in implant users has been successful with concurrent COC use.^33^ Usage of COC has not been investigated alongside the LNG-IUD and this could be a viable option for female astronauts, potentially providing a top-tier contraceptive, with the additional benefits of add-back estrogen, which may reduce bone loss, an important issue for astronauts. Further research would be needed to see whether this strategy decreases initial irregular bleeding, breakthrough bleeding, or has any impact on BMD.

### Recommendations for spaceflight

The uniqueness of the spaceflight environment provides many challenges in conducting research. The number of subjects required by clinical studies cannot be matched by the number of current active female astronauts. Combining pharmacological data on the bioavailability of hormones during spaceflight with analog ground-based studies investigating menstrual suppression may provide the evidence required to trial LARCs during spaceflight. With longer-duration missions, the age at which female astronauts are undertaking spaceflight is increasing and the literature supports the LNG-IUD as the progestin component for hormone replacement therapy in peri-menopausal and menopausal women. Research is needed into the use of the LNG-IUD alongside oral estrogen and whether this influences BMD in this subpopulation of astronauts.

Resource limitations as well as the continuous number of days worked by military personnel are similar to conditions experienced by astronauts. Lessons learned from military studies dictate that education for all female personnel at recruitment would be extremely beneficial in these populations of women autonomously making decisions about menstrual suppression. It is ultimately the woman’s choice to suppress or not. Respecting this autonomy is important; however, options should be available to her should she decide to suppress in consideration of her working environment.

## Figures and Tables

**Table 1:** Therapeutic options for medically induced amenorrhea available for female astronauts

*Medication*	*Dosing*	*Frequency*	*Contraceptive failure rate*	*Amenorrhea rate*	*Mechanism of action for amenorrhea*	*Advantages*	*Disadvantages*
Continuous COC	Multiple preparations, tend to be 30–35 μg ethinyl estradiol pill in continuous use	Daily, without pill-free week	Continuous and cyclical use have similar contraceptive efficacy^34^ with typical use they have a 9% failure rate^26^	Up to 80% at the end of 1 year of continuous use^26^	Estrogen and progesterone suppress hormone production and follicle development, endometrium thinner than normal	• Long history and experience with continuous use in spaceflight • Protect BMD compared with DMPA/non-hormonal contraceptives • Can be stopped immediately if required • Decreased risk of ovarian, endometrial, and colorectal cancer, iron deficiency anemia, benign breast disease, functional ovarian cysts, pre-menstrual symptoms, and dysmenorrhea^35^ • No impact on breast cancer rate	• Daily compliance required—potential issue with worldwide travel and training schedule • Variable duration of menstrual suppression • BTB particularly in initial phase • Hormonal side effects (estrogenic and progestogenic): migraine, VTE, stroke, liver problems, depression, glucose impairment, altered lipid metabolism, and vaginal infections • Estrogen is contraindicated in some women • Medication stability problematic on multi-year missions
Progestin-containing intrauterine device	LNG release 20 μg per day, e.g., Mirena (LNG-IUD)	Every 5 years	1-year failure rate is 0.2%^8^	Up to 80% at 1 year of use^8^ 62% at 2 years in peri-menopausal women for endometrial protection during HRT use^27^	LNG downregulates endometrial estrogen and progesterone receptors making endometrium insensitive to circulating estradiol, i.e., strong anti-proliferative effect. High local drug exposure to the uterine cavity leads to low LNG levels in serum (gradient of endometrium to serum >1,000-fold), leading to minimal systemic side effects^36^	• Long acting, i.e., no need to remember daily medication • No effect on BMD in femur and lumbar spine at 2 years^37^ • Top tier contraceptive efficacy • Treatment of choice in medical conditions including HMB, iron deficiency anemia, endometrial hyperplasia, endometriosis, adenomyosis, fibroids, and dysmenorrhea^35^ • Proliferative endometrium much lower in LNG-IUD versus oral progesterone, therefore rate of amenorrhea increased with time^38^ • Fertility regained after removal • Can be used in succession for continual benefits • Discontinuation rates much lower than COC use^38^ • No upmass or trash to dispose • Obviates long-duration medication stability issue	• Perforation (rate 1:1,000 insertions up to 9:1,000 insertions.^39–41^ Occur mainly in first year of use. May be related to skill of the operator or undetected uterine abnormalities • Expulsion rate between 2 and 10% in the first year^41^ • Infection (0.51% in the first year)^42^ • Variable duration of initial menstrual suppression due to BTB • Initial expense • Insertion-related pain/discomfort • Hormonal side effects—progesterone related (reduce with time)
Progestin-containing subdermal implant	Etonorgestrel, e.g., Nexplanon Levonorgestrel, e.g., Jadelle	3 years (Nexplanon) or 5 years (Jadelle)	1-year failure rate is 0.05%^41^	Varies between 11% in first 90 days up to 41.25% at 3 years^8,43–45^	Inhibits ovulation by suppressing hormone production and follicle development rendering the endometrium thinner than normal (such as COC)^46^	• Long acting with no need to remember daily medication • No effect on BMD at 2 years compared with non-hormonal contraceptives^46^ • Fertility regained after removal • Can be used in succession for continual benefits • No upmass or trash to dispose • Obviates long-duration medication stability issue	• Subdermal implant may be palpable on arm • Potential scarring at the site of insertion • Insertion discomfort • Variable length of time of menstrual suppression with initial BTB (27–51.25% irregular bleeding pattern)^44,45^ • Initial cost • Hormonal side effects (progesterone related)

Abbreviations: BMD, bone mineral density; BTB, breakthrough bleeding; COC, combined oral contraceptive pill; DMPA, depot medroxyprogesterone acetate injections; HMB, heavy menstrual bleeding; LNG, levonorgestrel; LNG-IUD, levonorgestrel intrauterine device; VTE, venous thromboembolism.

## References

[bib1] Thomas, S. L. & Ellertson, C. Nuisance or natural and healthy: should monthly menstruation be optional for women? Lancet 355, 922–924 (2000). 10752720 10.1016/S0140-6736(99)11159-0

[bib2] Glasier A. F. et al. Amenorrhea associated with contraception—an international study on acceptability. Contraception 67, 1–8 (2003). 12521650 10.1016/s0010-7824(02)00474-2

[bib3] Andrist L. C. et al. Women's and providers' attitudes toward menstrual suppression with extended use of oral contraceptives. Contraception 70: 359–363 (2004). 15504373 10.1016/j.contraception.2004.06.008

[bib4] Frederick, C. E., Edelman, A., Carlson, N. E., Rosenberg, K. D. & Jensen, J. T. Extended-use oral contraceptives and medically induced amenorrhea: attitudes, knowledge and prescribing habits of physicians. Contraception 84, 384–389 (2011). 21920194 10.1016/j.contraception.2011.02.004

[bib5] Lin, K. & Barnhart, K. The clinical rationale for menses-free contraception. J. Womens Health (Larchmt) 16, 1171–1180 (2007). 17937570 10.1089/jwh.2007.0332

[bib6] Hillard, P. A. Menstrual suppression: current perspectives. Int. J. Womens Health 6, 631–637 (2014). 25018654 10.2147/IJWH.S46680 PMC4075955

[bib7] Jones, J. A., Jennings, R. T., Baker, E. S.Renal, In: Biomedical Results of the Space Shuttle Program (eds Risin D. & Stepaniak P. C.) 141–155 (National Aeronautics and Space Administration, Lyndon B. Johnson Space Center, Houston, Texas, USA, 2013).

[bib8] ACOG Practice Bulletin No. 110: noncontraceptive uses of hormonal contraceptives. Obstet. Gynecol. 115, 206–218 (2010). 20027071 10.1097/AOG.0b013e3181cb50b5

[bib9] Makuch, M. Y., D Osis, M. J., de Padua, K. S. & Bahamondes, L. Use of hormonal contraceptives to control menstrual bleeding: attitudes and practice of Brazilian gynecologists. Int. J. Womens Health 5, 795–801 (2013). 24399887 10.2147/IJWH.S52086 PMC3876489

[bib10] Jones, J., Mosher, W. & Daniels, K. Current contraceptive use in the United States, 2006-2010, and changes in patterns of use since 1995. Natl Health Stat. Rep. 60, 1–25 (2012). 24988814

[bib11] Kavanaugh, M. L., Jerman, J. & Finer, L. B. Changes in use of long-acting reversible contraceptive methods among U.S. women, 2009-2012. Obstet. Gynecol. 126, 917–927 (2015). 26444110 10.1097/AOG.0000000000001094 PMC4946164

[bib12] O'Neil-Callahan, M., Peipert, J. F., Zhao, Q., Madden, T. & Secura, G. Twenty-four-month continuation of reversible contraception. Obstet. Gynecol. 122, 1083–1091 (2013). 24104781 10.1097/AOG.0b013e3182a91f45 PMC4012225

[bib13] Powell-Dunford, N., Cuda, A. S., Moore, J. L., Crago, M. S. & Deuster, P. A. Menstrual suppression using oral contraceptives: survey of deployed female aviation personnel. Aviat. Space Environ. Med. 80, 971–975 (2009). 19911522 10.3357/asem.2566.2009

[bib14] Trego, L. L. & Jordan, P. J. Military women's attitudes toward menstruation and menstrual suppression in relation to the deployed environment: development and testing of the MWATMS-9 (short form). Womens Health Issues 20, 287–293 (2010). 20627773 10.1016/j.whi.2010.03.002

[bib15] Powell-Dunford N. C. et al. Menstrual suppression for combat operations: advantages of oral contraceptive pills. Womens Health Issues 2011; 21: 86–91. 21185993 10.1016/j.whi.2010.08.006

[bib16] Crabb, S. L. Contraception counselling of female soldiers in primary healthcare facilities. J. R. Army Med. Corps 161, 109–111 (2015). 25034537 10.1136/jramc-2014-000280

[bib17] Secura, G. M., Allsworth, J. E., Madden, T., Mullersman, J. L. & Peipert, J. F. The Contraceptive CHOICE Project: reducing barriers to long-acting reversible contraception. Am. J. Obstet. Gynecol. 203, 115, e1-7 (2010). 20541171 10.1016/j.ajog.2010.04.017 PMC2910826

[bib18] Talukdar N. et al. Effect of long-term combined oral contraceptive pill use on endometrial thickness. Obstet. Gynecol. 120, 348–354 (2012). 22825095 10.1097/AOG.0b013e31825ec2ee

[bib19] Krassas, G. E. & Papadopoulou, P. Oestrogen action on bone cells. J. Musculoskelet Neuronal Interact. 2, 143–151 (2001). 15758462

[bib20] Dimitriou L. et al. Bone mineral density, rib pain and other features of the female athlete triad in elite lightweight rowers. BMJ Open 4, e004369 (2014). 10.1136/bmjopen-2013-004369 PMC3927798 24523427

[bib21] Lopez, L. M., Grimes, D. A., Schulz, K. F. & Curtis, K. M. Steroidal contraceptives: effect on bone fractures in women. Cochrane Database Syst. Rev. CD006033 (2009). 10.1002/14651858.CD006033.pub3 19370623

[bib22] Berenson, A. B., Rahman, M., Breitkopf, C. R. & Bi, L. X. Effects of depot medroxyprogesterone acetate and 20-microgram oral contraceptives on bone mineral density. Obstet. Gynecol. 112, 788–799 (2008). 18827121 10.1097/AOG.0b013e3181875b78 PMC2745348

[bib23] Berenson, A. B., Breitkopf, C. R., Grady, J. J., Rickert, V. I. & Thomas, A. Effects of hormonal contraception on bone mineral density after 24 months of use. Obstet. Gynecol. 103, 899–906 (2004). 15121563 10.1097/01.AOG.0000117082.49490.d5

[bib24] Weisberg, E., Bateson, D., McGeechan, K. & Mohapatra, L. A three-year comparative study of continuation rates, bleeding patterns and satisfaction in Australian women using a subdermal contraceptive implant or progestogen releasing-intrauterine system. Eur. J. Contracept. Reprod. Health Care 19, 5–14 (2014). 24229367 10.3109/13625187.2013.853034

[bib25] Sordal, T., Inki, P., Draeby, J., O'Flynn, M. & Schmelter, T. Management of initial bleeding or spotting after levonorgestrel-releasing intrauterine system placement: a randomized controlled trial. Obstet. Gynecol. 121, 934–941 (2013). 23635728 10.1097/AOG.0b013e31828c65d8

[bib26] Curtis, K. M., Bauer, U., Barfield, W. & Prevention, N. C. C. D. US Selected Practice Recommendations for Contraceptive Use, 2013 Adapted from the World Health Organization Selected Practice Recommendations for Contraceptive Use, 2nd Edition. MMWR Recomm. Rep. 62, 1–59 (2013). 23784109

[bib27] Boon, J., Scholten, P. C., Oldenhave, A. & Heintz, A. P. M. Continuous intrauterine compared with cyclic oral progestin administration in perimenopausal HRT. Maturitas 46, 69–77 (2003). 12963171 10.1016/s0378-5122(03)00163-4

[bib28] FSRH. CEU Guidance 2015 Problematic Bleeding with Hormonal Contraception. 1–28 (2015).

[bib29] Nappi, R. E., Kaunitz, A. M. & Bitzer, J. Extended regimen combined oral contraception: A review of evolving concepts and acceptance by women and clinicians. Eur. J. Contracept. Reprod. Health Care 1–9 (2015); e-pub ahead of print. 10.3109/13625187.2015.1107894 PMC4841029 26572318

[bib30] Bednarek, P. H. & Jensen, J. T. Safety, efficacy and patient acceptability of the contraceptive and non-contraceptive uses of the LNG-IUS. Int. J. Womens Health 1, 45–58 (2010). 21072274 10.2147/ijwh.s4350 PMC2971715

[bib31] Cibula D. et al. Hormonal contraception and risk of cancer. Hum. Reprod. Update 6, 631–650 (2010). 10.1093/humupd/dmq022 20543200

[bib32] ACOG Committee on Practice Bulletins–Gynecology. ACOG Practice Bulletin No. 117: Gynecologic care for women with human immunodeficiency virus. Obstet. Gynecol. 16, 1492–1509 (2010). 10.1097/AOG.0b013e3182054cae 21099636

[bib33] Abdel-Aleem, H., d'Arcangues, C., Vogelsong, K. M., Gaffield, M. L. & Gulmezoglu, A. M. Treatment of vaginal bleeding irregularities induced by progestin only contraceptives. Cochrane Database Syst. Rev. CD003449 (2013). 10.1002/14651858.CD003449.pub4 23828544

[bib34] Edelman A., Micks E., Gallo M. F., Jensen J. T., Grimes D. A. Continuous or extended cycle vs cyclic use of combined hormonal contraceptives for contraception. Cochrane Database Syst. Rev. CS004895 (2014). 10.1002/14651858.CD004695.pub3 PMC6837850 25072731

[bib35] Fraser, I. S. Added health benefits of the levonorgestrel contraceptive intrauterine system and other hormonal contraceptive delivery systems. Contraception 87, 273–279 (2013). 23040129 10.1016/j.contraception.2012.08.039

[bib36] Bayer. Mirena Data Sheet. Product Insert 1–15 (2014); http://www.medsafe.govt.nz/profs/datasheet/m/Mirenaius.pdf.

[bib37] Yang, K. Y., Kim, Y. S., Ji, Y. I. & Jung, M. H. Changes in bone mineral density of users of the levonorgestrel-releasing intrauterine System. J. Nippon Med. Sch. 79, 190–194 (2012). 22791119 10.1272/jnms.79.190

[bib38] Somboonporn, W., Panna, S., Temtanakitpaisan, T., Kaewrudee, S. & Soontrapa, S. Effects of the levonorgestrel-releasing intrauterine system plus estrogen therapy in perimenopausal and postmenopausal women: systematic review and meta-analysis. Menopause 18, 1060–1066 (2011). 21720280 10.1097/gme.0b013e31821606c5

[bib39] Van Houdenhoven, K., van Kaam, K. J., van Grootheest, A. C., Salemans, T. H. & Dunselman, G. A. Uterine perforation in women using a levonorgestrel-releasing intrauterine system. Contraception 73, 257–260 (2006). 16472566 10.1016/j.contraception.2005.08.013

[bib40] Margarit, L. M., Griffiths, A. N. & Vine, S. J. Management of levonorgestrel-releasing intrauterine system (LNG-IUS) uterine perforation. J. Obstet. Gynaecol. 24, 586–587 (2004). 15369954 10.1080/01443610410001722815

[bib41] American College of Obstetricians and Gynecologists ACOG Practice Bulletin No. 121: long-acting reversible contraception: implants and intrauterine devices. Obstet. Gynecol. 18, 184–196 (2011). 10.1097/AOG.0b013e318227f05e 21691183

[bib42] Eisenberg D. L. et al. Three-year efficacy and safety of a new 52-mg levonorgestrel-releasing intrauterine system. Contraception 2015; 92, 10–16. 25934164 10.1016/j.contraception.2015.04.006

[bib43] Hubacher, D., Lopez, L., Steiner, M. J. & Dorflinger, L. Menstrual pattern changes from levonorgestrel subdermal implants and DMPA: systematic review and evidence-based comparisons. Contraception 80, 113–118 (2009). 19631785 10.1016/j.contraception.2009.02.008

[bib44] Bhatia, P., Nangia, S., Aggarwal, S. & Tewari, C. Implanon: subdermal single rod contraceptive implant. J. Obstet. Gynaecol. India 61, 422–425 (2011). 22851825 10.1007/s13224-011-0066-z PMC3295879

[bib45] Gezginc, K., Balci, O., Karatayli, R. & Colakoglu, M. C. Contraceptive efficacy and side effects of Implanon. Eur. J. Contracept. Reprod. Health Care 12, 362–365 (2007). 17853166 10.1080/13625180701548040

[bib46] Hohmann, H. Examining the efficacy, safety, and patient acceptability of the etonogestrel implantable contraceptive. Patient Prefer. Adherence 3, 205–211 (2009). 19936163 10.2147/ppa.s4299 PMC2778430

